# Potential Selection Biases

**DOI:** 10.1289/ehp.113-1253785

**Published:** 2005-03

**Authors:** Carl-Gustaf Bornehag, Jan Sundell, Charles J. Weschler, Torben Sigsgaard

**Affiliations:** Public Health Sciences, Karlstad University, Karlstad, Sweden, E-mail: carl-gustaf.bornehag@kau.se; Technical University of Denmark, Lyngby, Denmark; Aarhus University, Aarhus, Denmark

In a recent article we reported an association [adjusted odds ratios (ORs) in the range of 2–3] between the concentrations of specific phthalates in dust from children’s bedrooms and doctor-diagnosed disease among children (Bornehag et al. 2004). This study has been accused of “selection bias problems” by the American Chemistry Council Phthalate Esters Panel (American Chemistry Council 2004) and the European Council for Plasticisers and Intermediates (2004).

A major criticism of the study is based on the assumption that families with allergic members change their flooring from carpets to a hard floor [e.g., polyvinyl chloride (PVC)]. If this is true, our findings would be biased (i.e., families with allergic members would have relatively more PVC flooring and would thus be exposed to higher concentrations of phthalates). However, very few families in Sweden today have wall-to-wall carpets; in total, 1% of the homes in this study reported having such carpets, two cases and two controls. Given that Swedish homes already have hard floors, there is no obvious reason why allergic families would change from one hard floor (e.g., wood) to another hard floor (e.g., PVC).

Still, there is some bias in the study. The case–control study in question is based on a cross-sectional baseline survey. The baseline questionnaire showed that there were no indications of selection bias among cases concerning self-reported flooring materials in the home (Bornehag CG, Sundell J, Sigsgaard T, Janson S, unpublished data). However, among controls, a main difference was a significant over-representation of wood flooring among participating families compared with non-participating families. Furthermore, on the basis of inspectors’ observations, parents sometimes misclassified the type of flooring material in their home. Quite often parents classified PVC as linoleum or cork.

Presuming the misclassification of linoleum and cork is similar among participating and nonparticipating families, then the distribution of flooring materials can be recalculated. The distribution of PVC flooring after such a recalculation becomes 59.8% (including cases), 61.2% (nonparticipating cases), 51.1% (included controls), and 59.5% (nonparticipating controls). Consequently, cases report PVC flooring slightly more often than controls. However, regarding hard flooring, we found no bias between the groups because 99% of the families had hard floors.

Even if PVC-flooring is associated with phthalates in dust, it is not simply a proxy for phthalate exposure. Several observations support such a statement. First, our study indicated that there are other significant indoor sources for phthalates because, in the absence of PVC flooring, the dust concentrations of plastisizers are still significant (Bornehag CG, Sundell J, Lundgren B, Weschler CJ, Sigsgaard T, Hagerhed-Engman L, unpublished data). Second, the association between doctor-diagnosed disease and the concentration of specific phthalates in dust was much stronger than the association between disease and PVC flooring (Bornehag et al. 2004). Third, the correlation between the concentration of different phthalates was rather weak (*r* < 0.35) (Bornehag et al. 2004). There are still other observations that indicate that the reported association is not due to selection bias. Fourth, an under-representation of about 9% of PVC flooring among controls cannot explain reported ORs in the range of 2–3. (However, the earlier reported association between PVC flooring and case status, OR 1.59, is overestimated because there is selection bias regarding PVC flooring among controls.) Fifth, when we included in the analyses only buildings with PVC in the child’s bedroom, the association between butyl benzyl phthalate (BBzP) in dust and rhinitis and eczema remained (Bornehag et al. 2004). In such a restricted analysis, the potential selection bias has been eliminated. Finally, we found different associations for different phthalates: di(2-ethylhexyl phthalate (DEHP) was associated with asthma; BBzP was associated with rhinitis and eczema; and DnBP was not associated with asthma or allergies.

In summary, for the reasons stated above, we judge that our reported association between phthalates in dust and asthma/allergic symptoms among children is not a consequence of either selection bias or active avoidance of specific flooring materials because of allergic disease in the family.
